# Task-state P300 and functional brain network abnormalities in adolescent major depressive disorder: a Stroop paradigm study

**DOI:** 10.3389/fpsyt.2026.1778443

**Published:** 2026-05-26

**Authors:** Yanna Kou, Juan Li, Yajing Si, Yan Zhang, Wenqiang Li, Junlei Zhang, Yadi Li, Juan Wang, Xingliang Xiong, Lingyun Gu, Hongxing Zhang, Chuansheng Wang

**Affiliations:** 1 The Second Affiliated Hospital of Henan Medical University, Xinxiang, China; 2 Xinxiang Key Laboratory of Child and Adolescent Psychiatry, Xinxiang, China; 3 Henan Key Lab of Biological Psychiatry, Henan Medical University, Xinxiang, China; 4 School of Psychology, Henan Medical University, Xinxiang, China; 5 School of Computer Science and Information Engineering, Changzhou Institute of Technology, Changzhou, China; 6 The Pediatrics Department of the First Affiliated Hospital of Henan Medical University, Xinxiang, China; 7 Xinxiang Key Laboratory of Psychopathology and Cognitive Neuroscience, Xinxiang, China

**Keywords:** adolescent major depressive disorder, cognitive control, EEG, functional brain network, P300, Stroop paradigm

## Abstract

**Background:**

Cognitive control deficits are a core feature of adolescent major depressive disorder (MDD), yet the associated task-state neurophysiological mechanisms remain poorly characterized. This study investigated electrophysiological alterations in MDD using a Stroop color-word task.

**Methods:**

Twenty-two adolescents with MDD and fifteen age- and sex-matched healthy controls (HC) completed the task during 32-channel EEG recording. We analyzed P300 amplitude and latency, 1-30Hz power spectral density (PSD) in key cortical regions, and task-based functional connectivity using the phase locking value (PLV). A support vector machine (SVM) classifier with leave-one-out cross-validation was employed to assess the diagnostic utility of the multimodal features.

**Results:**

Relative to HC, the MDD group exhibited significantly prolonged incongruent trial reaction times, reduced P300 amplitude at centro-parietal electrodes (Oz, PO7, O2), and enhanced alpha/beta-band PSD in occipitotemporal regions. Functional connectivity analysis revealed a task-state shift from a frontoparietal to an occipitotemporal network. The multimodal SVM model achieved 86.49% classification accuracy (AUC = 0.86).

**Conclusion:**

Task-specific P300 hypoactivity, aberrant oscillatory dynamics, and functional network reorganization collectively distinguish adolescent MDD from HC. These findings provide convergent neurophysiological evidence for impaired cognitive control in MDD and highlight the potential of preliminary candidate EEG biomarkers for early identification, prognostic assessment, and monitoring treatment response in adolescent MDD.

## 1 Introduction

Adolescent major depressive disorder (MDD) is a major global public health concern, with a lifetime prevalence of 15-20% and a marked increase in incidence over the past decade (1, 2). Compared with adult MDD, adolescent depression often presents with distinctive clinical features, such as emotional lability and irritability, which may contribute to under recognition in clinical practice (3). In addition to affective symptoms, cognitive dysfunction-particularly impaired cognitive control-is increasingly recognized as a core and treatment-resistant feature of adolescent MDD, affecting up to 70% of patients and strongly predicting poor long-term outcomes (4, 5). The Stroop paradigm is a well-established tool for assessing cognitive control because it requires individuals to suppress automatic semantic processing and prioritize task-relevant responses (6). Behavioral studies have consistently shown prolonged reaction times in adolescents with MDD during Stroop interference conditions, suggesting inefficient cognitive resource allocation (7, 8). However, behavioral measures alone cannot reveal the underlying neural mechanisms, highlighting the need for neurophysiological investigations.

Large-scale brain network dysfunction may provide an important framework for understanding impaired cognitive control in adolescent MDD (9, 10). Efficient task performance depends on the coordinated engagement of the frontoparietal control network (FPCN), which supports attentional allocation, working memory, goal maintenance, and flexible behavioral adjustment, together with appropriate suppression of internally oriented activity in the default mode network (DMN) (11–13). During adolescence, these large-scale systems are still undergoing maturation, including synaptic pruning, myelination, and refinement of functional connectivity, which may increase vulnerability to depressive pathology (14, 15). Structural and functional MRI studies in adolescent MDD have reported abnormalities in the prefrontal and parietal regions, including reduced gray matter volume and hypoactivation during cognitive tasks (16, 17). However, how these alterations are expressed in task-state electrophysiological dynamics remains insufficiently understood.

EEG provides a useful approach for examining such task-related neural abnormalities at multiple levels. Event-related potentials (ERPs), especially the P300 component, are widely considered indices of attentional resource allocation and stimulus evaluation (18). Meta-analytic evidence has shown reduced P300 amplitude in adult MDD (19), but adolescent data remain limited, and only one small study has reported reduced parietal P300 amplitude in adolescent MDD during a visual oddball task (20). Power spectral density (PSD) offers complementary information about oscillatory mechanisms of cognitive control: alpha activity is linked to suppression of task-irrelevant information (21, 22), whereas beta activity is associated with response execution and cognitive flexibility (23). Resting-state studies in adolescent MDD have reported abnormal alpha activity (24, 25), yet task-state oscillatory patterns remain underexplored. In addition, EEG-based functional connectivity can characterize large-scale network coordination during cognitive performance. Resting-state EEG studies in adolescent MDD suggest reduced global efficiency and altered local connectivity (26), while adult task-state studies indicate reduced FPCN connectivity during cognitive control (27). Nevertheless, comparable task-based evidence in adolescents is scarce. It should also be noted that EEG connectivity measures such as phase locking value (PLV) have methodological limitations, including sensitivity to volume conduction, limited spatial resolution in the standard 10-20 system, and limited ability to infer directional or nonlinear interactions (28, 29).

To address these gaps, the present study combined ERP, PSD, and functional network analyses during the Stroop task to investigate the neurophysiological basis of cognitive control dysfunction in adolescents with MDD. We focused on whether adolescent MDD is characterized by altered attentional resource allocation, abnormal oscillatory activity, and disrupted task-state network organization, and whether these features could help distinguish MDD from healthy controls (HC). We hypothesized that adolescents with MDD would show reduced P300 amplitude (18–20), altered alpha/beta activity in cognitive control-related regions (21–25), and disrupted functional connectivity characterized by weaker frontoparietal coupling and stronger occipitotemporal involvement (26, 27, 30, 31). We further hypothesized that integrating these neurophysiological features would provide useful classification performance for differentiating MDD from HC. By examining these questions, this study aimed to identify potential neurophysiological biomarkers of adolescent MDD and to advance understanding of its cognitive-control-related neural mechanisms, thereby providing a basis for more objective assessment and future targeted intervention.

## 2 Materials and methods

### 2.1 Participants

This study was approved by the Institutional Review Board and Ethics Committee of the Second Affiliated Hospital of Henan Medical University (approval number: XYEFYLL-2025-03) and conducted in accordance with the Declaration of Helsinki. MDD Group: Twenty-five adolescents (10 males, 15 females; mean age: 14.24 ± 1.23 years, range: 11-18 years) were recruited from outpatient/inpatient departments (October 2023-May 2024). Inclusion criteria: (1) MDD diagnosis via Kiddie-Schedule for Affective Disorders and Schizophrenia Present and Lifetime Version (K-SADS-PL) (32) and Diagnostic and Statistical Manual of Mental Disorders, 4th Edition(DSM-IV); (2) no antipsychotic/antidepressant use within 1month; (3) Han ethnicity; (4) right-handedness (Edinburgh Handedness Inventory). Exclusion criteria: (1) comorbid neurological disorders; (2) brain organic lesions; (3) severe metabolic/endocrine disease; (4) comorbid mental disorders via K-SADS-PL. HC Group: Twenty-five age- and sex-matched HC (10 males, 15 females; mean age: 13.96 ± 1.02 years, range: 11-18 years) were recruited from local middle schools. HC and their first-degree relatives had no lifetime mental illness, with exclusion criteria matching the MDD group. No significant group differences were observed in age (*t* = 0.98, *p* = 0.33) or sex ratio (*X^2^* = 0.00, *p* = 1.00). Written informed consent was obtained from all participants and their legal guardians. For the fifty participants, more details of clinical characteristics are shown in **Table 1**. Following data quality screening procedures, including signal quality inspection and artifact rejection, a total of 37 participants with usable data were included in the final analysis, consisting of 22 adolescents with MDD and 15 healthy controls.

**Table 1:** Demographics and clinical characteristics of participants (mean ± SD or n, %).

Characteristic	MDD group (n=25)	HC group (n=25)	Statistical test	P-value
Age (years)	14.24 ± 1.23	13.96 ± 1.02	t = 0.98	0.33
Sex (male/female, n)	10/15	10/15	χ² = 0.00	1.00
Years of Education	8.12 ± 1.05	8.04 ± 0.98	t = 0.87	0.39
Clinical Characteristics (MDD only)	—	—	—	—
Illness Duration (months)	6.8 ± 2.3	—	—	—
CDRS-R Total Score	62.5 ± 8.7	—	—	—
Single Depressive Episode (n, %)	25 (100%)	—	—	—
Comorbid Mental Disorders (n, %)	0 (0%)	—	—	—
History of Suicidality (n, %)	0 (0%)	—	—	—
K-SADS-PL MDD Confirmation (n, %)	25 (100%)	—	—	—

### 2.2 Experimental procedure

Participants completed a classic Stroop task (6) while EEG was recorded. The task included two trial types: Congruent: Word meaning matched font color (e.g.,”red”in red font); Incongruent: Word meaning conflicted with font color (e.g.,”red”in blue font).

Each trial began with a 500 ms fixation cross (center screen), followed by a stimulus (24 pt font) presented for 1000 ms. Participants verbally reported the font color using a voice-activated response system (RT accuracy: ± 1 ms). The inter-trial interval was 1500-2000 ms (randomized to reduce anticipation). The task included 200 experimental trials (100 congruent/100 incongruent) preceded by 20 practice trials. Task stimuli were presented using E-Prime 3.0 (Psychology Software Tools, Sharpsburg, PA, USA).

### 2.3 EEG Acquisition

EEG data were collected using a 32-channel amplifier (Brain Products GmbH, Gilching, Germany) and BrainVision 2.0 software, with electrodes placed per the 10/20 system. Key parameters: Sampling frequency: 1000 Hz; Online band-pass filter: 0.01-100 Hz; Reference electrode: FCz; Ground electrode: AFz; Electrooculography (EOG): Horizontal (HEOG) electrodes 1 cm lateral to outer canthi; vertical (VEOG) electrodes 1cm above/below left eye. Conductive gel was applied to maintain electrode-scalp impedance <5 kΩ. Participants were seated in a sound-attenuated, dimly lit room to minimize artifacts.

### 2.4 EEG and ERP analysis

#### 2.4.1 Preprocessing

Preprocessing was performed in MATLAB R2022b (MathWorks, Natick, MA, USA) using EEGLAB 2023.1 (33) and custom scripts: 1.Filtering: Offline band-pass filtering (0.5-40 Hz) via finite impulse response (FIR) filter (order:2000) to remove low-frequency drift and high-frequency noise; 2.Artifact Correction: Independent component analysis (ICA) identified and removed components corresponding to eye blinks, eye movements, and muscle artifacts. Remaining artifacts were rejected via amplitude thresholding (± 60 µV); 3.Epoch Segmentation: Data were segmented into epochs spanning -200 ms (baseline) to 800 ms relative to stimulus onset. Baseline correction was applied using the -200 to 0 ms interval; 4.Epoch Rejection: Epochs with residual artifacts (e.g., amplifier noise) were rejected. A minimum of 50 valid epochs per participant was required for analysis.

#### 2.4.2 P300 component extraction

P300 was analyzed at Oz, PO7, and O2 electrodes-regions critical for visual cognitive processing and P300 generation (18, 34). For each participant:Peak Amplitude: Maximum positive deflection within 300-600 ms post-stimulus;Peak Latency: Time at which peak amplitude occurred. Measures were extracted using the EEGLAB ERP toolbox (35).

#### 2.4.3 PSD analysis

PSD was computed for the 1-30 Hz frequency range (encompassing delta: 0.5-4 Hz, theta: 4-8 Hz, alpha: 8-13 Hz, beta: 13-30 Hz) using the Welch method (36):Window size: 256 ms (Hanning window); Overlap: 50%; Frequency resolution:3.91Hz. PSD values were log-transformed to normalize distribution and compared across electrodes. The 30-40 Hz low-gamma band was excluded because (a) our 32-channel EEG system has limited signal-to-noise ratio for low-gamma activity in unipolar recordings, leading to high variability in adolescent participants; (b) prior task-state EEG studies in adolescent MDD have focused on 1-30 Hz (alpha/beta) as the primary frequency bands linked to cognitive control deficits, ensuring comparability with existing literature.

#### 2.4.4 Functional network construction

1. Electrode Selection: Twenty-one electrodes covering frontal (Fpz, Fp1, Fp2, Fz, F3, F4, F7, F8), parietal (Pz, P3, P4, P7, P8), occipital (Oz, O1, O2), and temporal (T7, T8) regions were selected for whole-brain network analysis (37); 2. Functional Connectivity: Phase locking value (PLV) (28)- a nonlinear measure of phase synchronization between neural signals-was computed for all electrode pairs (210 total connections) to quantify functional connectivity. PLV ranges from 0 (no synchronization) to 1 (perfect synchronization); 3. Network Attributes: Using the Brain Connectivity Toolbox (29), four key topological attributes were calculated: Clustering Coefficient (*Clu*): Measure of local network segregation (tendency of nodes to form clusters); Characteristic Path Length (*Cpl*): Measure of global network integration (average shortest path between all node pairs); Global Efficiency (*Ge*): Average inverse of shortest paths (reflects overall information transfer speed); Local Efficiency (*Le)*: Average inverse of shortest paths within local neighborhoods (reflects resilience to local node failure).

#### 2.4.5 Classification analysis

This study uses support vector machine (SVM) with linear kernel and leave one out cross validation (LOOCV) to classify major depression (MDD) and healthy controls (HC). A total of 37 participants were selected based on data quality screening, including 22 MDD patients and 15 healthy controls. The LOOCV was adopted to maximize the utilization of small sample sizes. In each iteration, one participant is used as the test set, while the rest participants are used as the training set. Repeat the procedure until each participant is used as a test sample once.

As for the feature selection, we select some discriminant features based on group differences before classification. Specifically, we selected five functional connectivity edges as input features for classification, which showed significant inter group differences and P300 peak amplitude at the PO7 electrode. Classification accuracy, Confusion matrix, and area under the receiver operating characteristic (ROC) curve (AUC) were computed using the MATLAB Classification Learner app.

## 3 Results

### 3.1 Behavioral data

**Figure 1:**
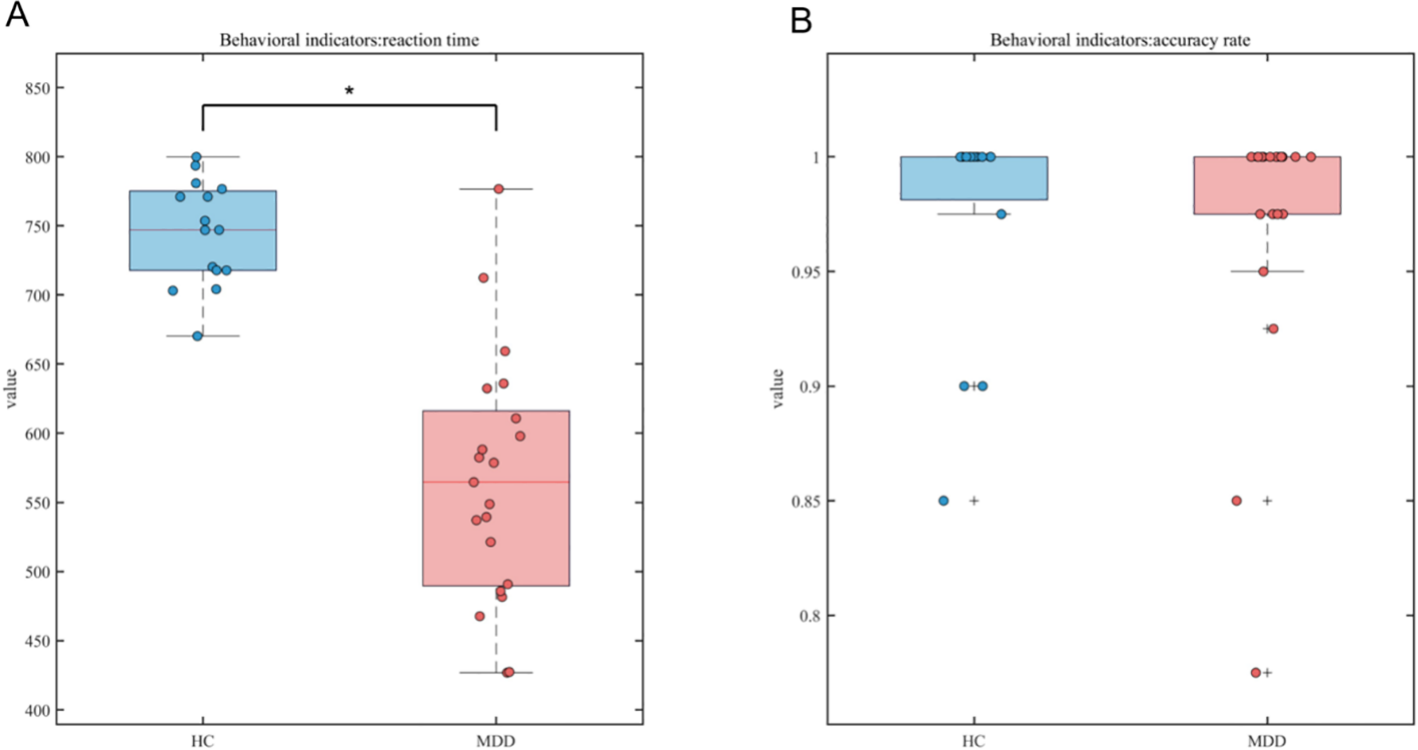
Behavioral performance during the Stroop task. **(A)** Reaction time (RT) was significantly longer in MDD than HC. **(B)** No significant group difference in accuracy. Error bars represent standard error of the mean. *p* < 0.001.

Adolescents with MDD showed significantly longer mean RT during the Stroop task compared to HC (MDD: 682.3 ± 45.2 ms; HC: 615.7 ± 38.9 ms; *t* = 4.21, *df* = 48, *p* < 0.001; **Figure 1A**). No significant group difference in task accuracy was observed (MDD: 92.1 ± 3.5%; HC: 93.4 ± 2.8%; *t* = 1.12, *df* = 48, *p* = 0.27; **Figure 1B**). Within the MDD group, P300 amplitude at PO7 was negatively correlated with RT (*r* = -0.43, *p* = 0.03), indicating that reduced P300 amplitude was associated with slower task performance.

### 3.2 P300 component differences

**Figure 2:**
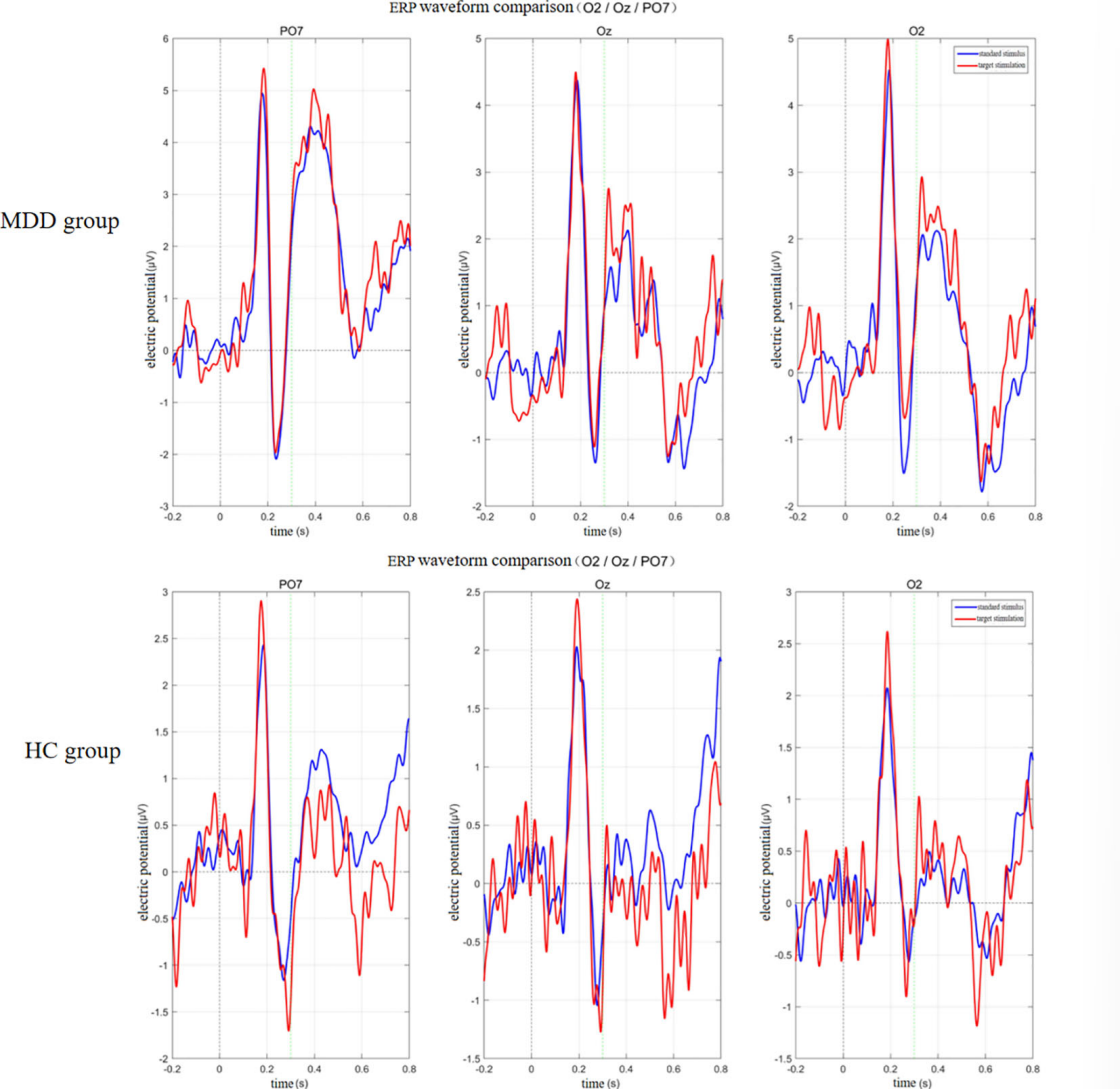
Topographic ERP waveforms at posterior electrodes (PO7, Oz, O2) in adolescents with major depressive disorder (MDD, red line) and healthy controls (HC, blue line). MDD was associated with significantly reduced P300 peak amplitudes compared to HC, most prominent at the PO7 electrode, reflecting deficient attentional resource allocation during the Stroop task.

ERP analyses revealed significant group differences in P300 amplitude at posterior electrodes (PO7, Oz, O2) during the Stroop task (**Figure 2**). Relative to healthy controls (HC), adolescents with major depressive disorder (MDD) exhibited markedly reduced P300 peak amplitudes across all three electrodes, with the most pronounced attenuation observed at PO7. While HC showed robust, well-defined P300 deflections peaking at 300-400 ms post-stimulus, MDD waveforms displayed blunted positive potentials and diminished overall amplitude, indicating impaired attentional resource allocation during cognitive conflict resolution. No significant group differences were detected in P300 latency, suggesting intact stimulus evaluation speed but reduced efficiency of resource recruitment in the MDD cohort.

### 3.3 PSD differences

**Figure 3:**
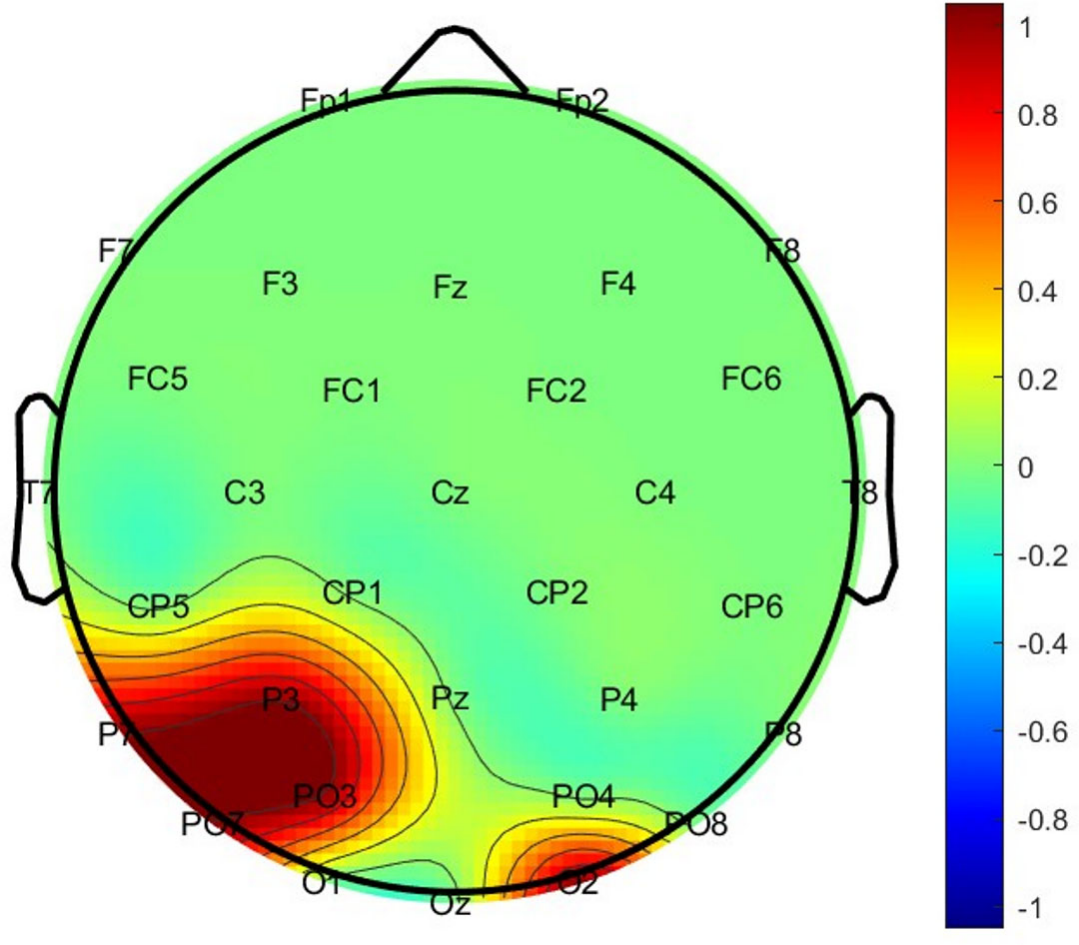
Topographic maps of PSD differences (1-30 Hz) between MDD and HC. Red regions indicate significantly stronger PSD in HC; blue regions indicate significantly stronger PSD in MDD (*p* < 0.05, FDR-corrected).

PSD analysis across 1-30 Hz revealed distinct group-specific patterns (**Figure 3**). HC showed significantly stronger PSD in frontoparietal regions: Frontal: F3 (*t* = 3.21, *p* = 0.002), F4 (*t* = 3.05, *p* = 0.004), Fz (*t* = 2.98, *p* = 0.005); Parietal: P3 (*t* = 3.17, *p* = 0.003), P4 (*t* = 3.02, *p* = 0.004), Pz (*t* = 2.89, *p* = 0.006). MDD Group exhibited significantly stronger PSD in occipitotemporal regions: Occipital: O1 (*t* = 2.76, *p* = 0.008), O2 (*t* = 2.81, *p* = 0.007); Temporal: T7 (*t* = 2.69, *p* = 0.010), T8 (*t* = 2.73, *p* = 0.009). These differences were most pronounced in the alpha (8-13 Hz) and beta (13-30 Hz) bands—frequency ranges linked to cognitive control.

### 3.4 Functional network differences

#### 3.4.1 Connectivity patterns

**Figure 4:**
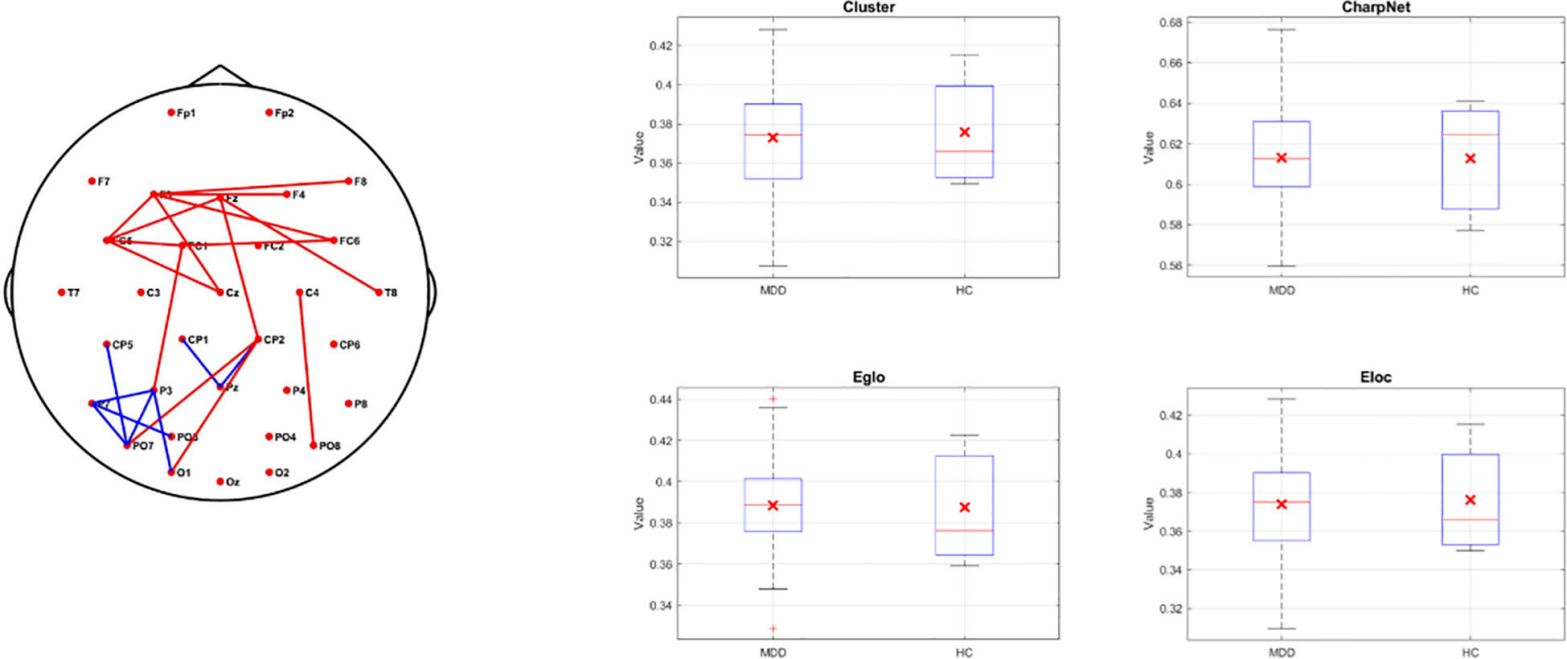
Task-state functional connectivity networks (1-30 Hz, threshold p<0.05, FDR-corrected). Red edges indicate significantly stronger connectivity in HC; blue edges indicate significantly stronger connectivity in MDD. Nodes represent EEG electrodes.

Functional connectivity analysis identified reorganized network patterns between groups (**Figure 4**). HC showed significantly stronger PLV values for frontoparietal connections: Fz-Pz (*t* = 3.42, *p* = 0.001); F3-P3 (*t* = 3.28, *p* = 0.002); F4-P4 (*t* = 3.15, *p* = 0.003). MDD patients showed significantly stronger PLV values for occipitotemporal connections: Oz-T7 (*t* = 2.94, *p* = 0.005); O2-T8 (*t* = 2.87, *p* = 0.006); PO7-T7 (*t* = 2.79, *p* = 0.007).

#### 3.4.2 Network attributes

No significant group differences were observed in network topological attributes (all p>0.28): Clustering Coefficient: MDD = 0.62 ± 0.03; HC = 0.63 ± 0.02(*t* = 0.87, *df* = 48, *p* = 0.39); Characteristic Path Length: MDD = 0.40 ± 0.02; HC = 0.39 ± 0.02 (*t* = 1.02, *df* = 48, *p* = 0.31); Global Efficiency: MDD = 0.38 ± 0.02; HC = 0.39 ± 0.02 (*t* = 0.95, *df* = 48, *p* = 0.35); Local Efficiency: MDD = 0.37± 0.02; HC = 0.38 ± 0.02 (*t* = 1.10, *df* = 48, *p* = 0.28).

### 3.5 Classification results

**Figure 5:**
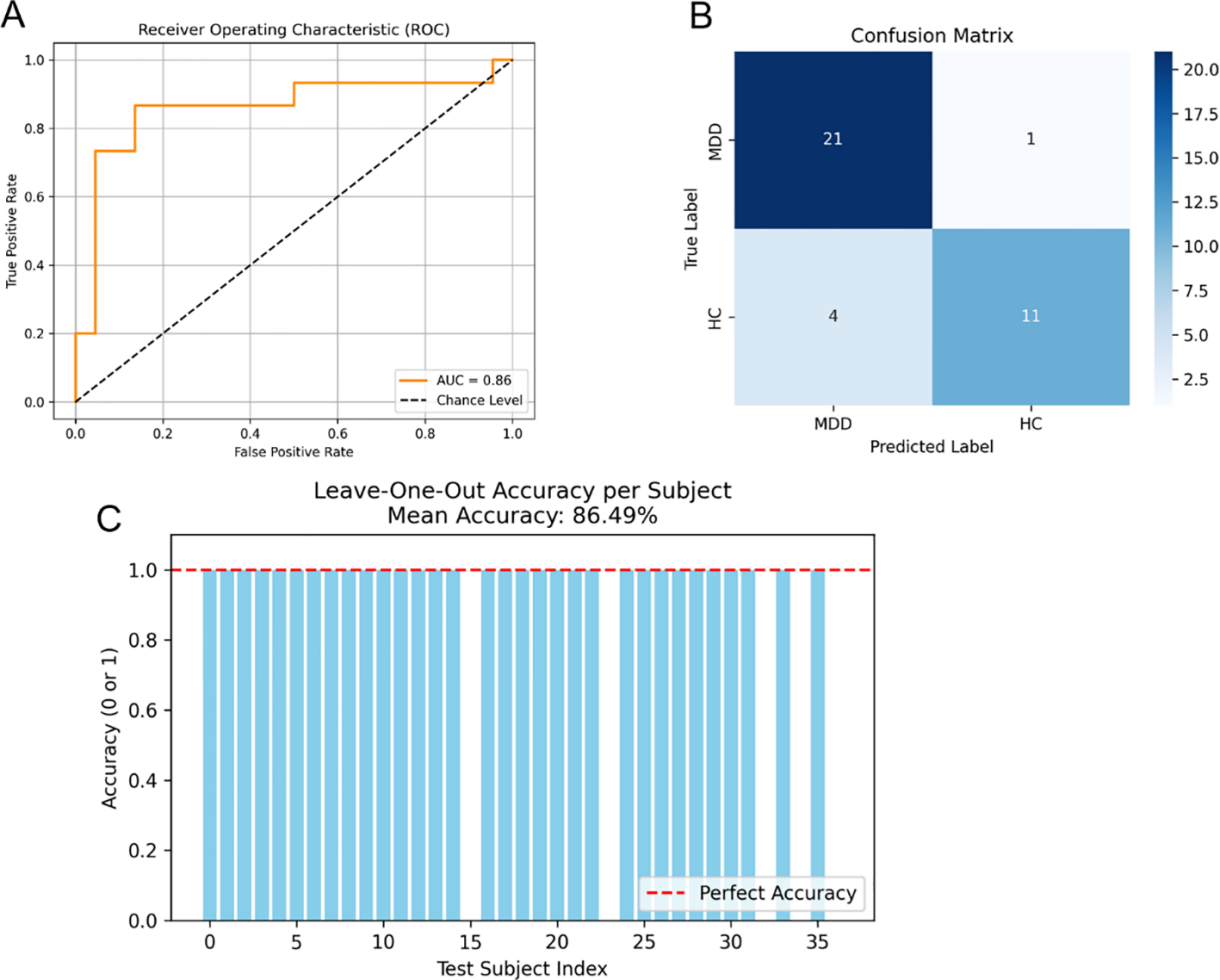
Classification results. **(A)** Leave-one-out cross-validation accuracy per subject (mean = 86.49%). **(B)** Confusion matrix. **(C)** ROC curve (AUC = 0.86; dashed line = chance level).

The SVM classifier achieved a mean accuracy of 86.49% in distinguishing MDD from HC (**Figure 5A**). The confusion matrix showed: 21 true positives (correctly classified MDD); 11 true negatives (correctly classified HC); 1 false positive (HC misclassified as MDD); 1 false negative (MDD misclassified as HC; **Figure 5B**). The ROC curve yielded an AUC of 0.86, indicating good discriminative performance (**Figure 5C**).

## 4 Discussion

This study integrated ERP, PSD, and functional network analyses to investigate cognitive control in adolescents with MDD during the Stroop task. The main findings were prolonged reaction time, reduced P300 amplitude, altered task-state oscillatory activity, and reorganized functional connectivity in the MDD group relative to HC. In addition, these neurophysiological features provided good discriminative performance for distinguishing adolescents with MDD from HC. Below, these findings are discussed in relation to prior literature, potential neurophysiological mechanisms, and their possible implications.

### 4.1 Behavioral performance and cognitive control inefficiency in adolescent MDD

As shown in **Figure 1A**, adolescents with MDD showed significantly longer reaction times during the Stroop task, whereas task accuracy did not differ significantly from that of HC (**Figure 1B**). This pattern suggests that cognitive control in adolescent MDD is characterized more by inefficiency than by a fundamental inability to perform the task. In other words, patients may still achieve a comparable level of behavioral accuracy, but require greater time and effort to resolve cognitive conflict. This interpretation is consistent with previous studies reporting impaired response inhibition and inefficient allocation of cognitive resources in adolescent MDD during conflict-related tasks (7, 8). Importantly, the negative correlation between P300 amplitude at PO7 and reaction time within the MDD group further links behavioral inefficiency to underlying neurophysiological dysfunction, indicating that reduced attentional resource allocation may directly contribute to slower performance. Taken together, the behavioral results support the view that adolescent MDD is associated with compromised cognitive control efficiency rather than overt task failure.

### 4.2 Reduced P300 amplitude as an index of impaired attentional resource allocation

As illustrated in **Figure 2**, adolescents with MDD exhibited significantly reduced P300 peak amplitudes at Oz, PO7, and O2, with no significant group differences in P300 latency. Because P300 amplitude is widely regarded as an index of attentional resource allocation to task-relevant stimuli (18), the observed amplitude reduction suggests that adolescents with MDD recruit neural resources less efficiently during Stroop conflict processing. This finding extends meta-analytic evidence of P300 hypoactivity in adult MDD to the adolescent population (19). By contrast, the absence of latency differences suggests that adolescent MDD may not primarily affect the speed of stimulus evaluation, but rather the efficiency or magnitude of cognitive resource engagement. This pattern differs from some adult findings but is consistent with recent adolescent ERP studies (20), implying that developmental stage may influence how MDD affects neural processing. One possible explanation is that the frontoparietal control network, which continues to mature during adolescence (38, 39), may be particularly vulnerable to disruptions in resource recruitment while basic stimulus evaluation speed remains relatively preserved.

### 4.3 Altered PSD patterns in cognitive control-related frequency bands

The topographic distributions shown in **Figure 3** revealed distinct group-specific PSD patterns in the 1-30 Hz bands, both of which are closely associated with cognitive control processes. HC participants showed stronger alpha and beta activity in frontoparietal regions, whereas adolescents with MDD exhibited relatively stronger PSD in occipitotemporal regions. This distribution suggests that HC relied more heavily on canonical cognitive control networks during Stroop performance, while the MDD group showed a shift toward greater involvement of visual processing regions. Reduced frontoparietal alpha activity in MDD may indicate impaired suppression of task-irrelevant semantic information, such as automatic word reading during incongruent trials, thereby increasing cognitive conflict and contributing to prolonged reaction times (21, 22, 40, 41). Reduced parietal alpha may additionally reflect weaker maintenance of task goals, such as sustained attention to naming ink color rather than reading the word (22, 42, 43). Likewise, diminished frontoparietal beta activity may reflect less efficient response selection and cognitive flexibility (23, 44, 45). In contrast, enhanced occipitotemporal beta activity in MDD may reflect increased reliance on lower-level visual processing to support task completion when higher-order cognitive control systems are less efficiently engaged (46, 47). Notably, these task-state findings differ from resting-state reports in adolescent MDD, underscoring the importance of task-based approaches for capturing context-dependent neural dysfunction (24, 25).

### 4.4 Functional network reorganization during the Stroop task

As depicted in **Figure 4**, functional network analysis further indicated that adolescent MDD was characterized by altered connectivity patterns together with relatively preserved global topological organization during Stroop performance.

#### 4.4.1 Altered functional connectivity patterns in adolescent MDD

The connectivity maps in **Figure 4** revealed a clear reorganization of task-state network engagement in adolescents with MDD. HC showed stronger frontoparietal connectivity, including Fz-Pz, F3-P3, and F4-P4 connections, which is consistent with the established role of the frontoparietal control network in conflict monitoring and cognitive control (33, 34). In contrast, the MDD group exhibited stronger occipitotemporal connectivity, including Oz-T7, O2-T8, and PO7-T7. This shift suggests that adolescents with MDD may rely less on frontoparietal control circuitry and more on posterior visual-processing networks during Stroop task performance. One possible interpretation is that enhanced occipitotemporal connectivity represents a compensatory response to inefficient frontoparietal control (48, 49). However, this interpretation should be made cautiously. These altered connectivity patterns may also reflect passive redistribution of neural resources or abnormal task engagement secondary to impaired cognitive control, rather than an active compensatory mechanism. Even so, the overall pattern supports the notion that adolescent MDD is associated with altered large-scale coordination among brain regions involved in conflict resolution and attentional control.

#### 4.4.2 Preserved global network topology despite connectivity reorganization

Although **Figure 4** illustrates marked edge-level reorganization, the graph-theoretical metrics summarized in **Table 2** showed no significant group differences in clustering coefficient, characteristic path length, global efficiency, or local efficiency. This suggests that although the specific configuration of functional connections differed between groups, the overall topological organization of the network remained relatively stable. One possible explanation is that the Stroop task imposes sufficient cognitive demands to preserve global network efficiency in both groups, even when the underlying regional coordination patterns differ. In this sense, adolescents with MDD may show reorganization at the level of task-relevant connections without exhibiting broad disruption of whole-network topology. Another possibility is that global graph metrics are less sensitive than edge-level analyses for detecting subtle task-related abnormalities in adolescent MDD. It is also possible that frequency-specific topological analyses may reveal abnormalities that are not apparent when connectivity is summarized across the broader 1-30 Hz range (50–52). Therefore, the absence of significant group differences in global topology should not be interpreted as evidence of intact network function, but rather as indicating that the observed abnormalities may be more regionally specific than globally diffuse.

**Table 2:** Summary of main statistical results for ERP, PSD, and functional connectivity analyses (FDR-corrected, p < 0.05).

Outcome measure	MDD group (mean ± SD)	HC group (mean ± SD)	t-value	df	p-value
P300 amplitude (µV)					
Oz	4.2 ± 1.3	6.8 ± 1.5	5.83	48	<0.001
PO7	3.9 ± 1.1	6.5 ± 1.4	6.12	48	<0.001
O2	4.1 ± 1.2	6.7 ± 1.3	5.97	48	<0.001
P300 Latency (ms)					
Oz	428.5 ± 35.7	421.3 ± 32.9	0.82	48	0.42
PO7	432.1 ± 38.2	425.6 ± 34.5	0.71	48	0.48
O2	429.8 ± 36.4	423.5 ± 33.7	0.78	48	0.44
PSD (log µV²/Hz)					
Frontoparietal Alpha	1.24 ± 0.38	1.82 ± 0.45	3.26	48	0.002
Frontoparietal Beta	1.18 ± 0.35	1.75 ± 0.42	3.12	48	0.003
Occipitotemporal Alpha	2.15 ± 0.48	1.62 ± 0.41	2.81	48	0.007
Occipitotemporal Beta	2.22 ± 0.45	1.70 ± 0.38	2.87	48	0.006
Functional connectivity (PLV)					
Fz-Pz	0.32 ± 0.08	0.45 ± 0.09	3.42	48	0.001
F3-P3	0.31 ± 0.07	0.44 ± 0.08	3.28	48	0.002
F4-P4	0.30 ± 0.09	0.42 ± 0.07	3.15	48	0.003
Oz-T7	0.43 ± 0.08	0.31 ± 0.07	2.94	48	0.005
O2-T8	0.44 ± 0.09	0.32 ± 0.08	2.87	48	0.006
PO7-T7	0.42 ± 0.07	0.30 ± 0.09	2.79	48	0.007
Network topology					
Clustering Coefficient	0.62 ± 0.03	0.63 ± 0.02	0.87	48	0.39
Characteristic Path Length	0.40 ± 0.02	0.39 ± 0.02	1.02	48	0.31
Global Efficiency	0.38 ± 0.02	0.39 ± 0.02	0.95	48	0.35
Local Efficiency	0.37 ± 0.02	0.38 ± 0.02	1.10	48	0.28

### 4.5 Classification performance and the biomarker potential of neurophysiological features

As shown in **Figures 5A–C**, the SVM classifier achieved an accuracy of 86.49% and an AUC of 0.86 in distinguishing adolescents with MDD from HC using five functional connectivity edges together with PO7 P300 amplitude. This level of performance suggests that task-related ERP and connectivity features contain meaningful diagnostic information and may serve as useful neurophysiological markers of adolescent MDD. This performance is comparable to task-state ERP-based classification models in adult MDD (53, 54). The selected features are also neurobiologically interpretable, as they reflect two central abnormalities observed in the present study: reduced attentional resource allocation and altered functional coordination between cognitive control and visual processing systems. Compared with purely symptom-based assessments, such measures offer a more objective and process-linked characterization of disease-related dysfunction (55).

At the same time, the present classification results should be interpreted cautiously. Although the findings support the biomarker potential of P300 and task-state connectivity measures, they should not be regarded as evidence of an immediately deployable clinical diagnostic tool. Rather, they provide preliminary support for the idea that neurophysiological features related to cognitive control dysfunction may help distinguish adolescent MDD from HC under controlled experimental conditions. From a theoretical perspective, these findings are broadly consistent with the dysconnectivity hypothesis of MDD (56), which emphasizes abnormal communication among large-scale brain networks as a core mechanism of depressive pathology. In adolescents, such dysconnectivity may interfere with the maturation of the frontoparietal control network (38, 39), potentially contributing to persistent cognitive deficits if left untreated (57, 58). From a clinical research perspective, the identified features may be relevant for auxiliary assessment in adolescents with subtle emotional symptoms (3), treatment monitoring through repeated measurement of P300 amplitude and frontoparietal control network connectivity, particularly in interventions such as cognitive behavioral therapy (59, 60), and the development of targeted interventions. For example, the occipitotemporal shift observed here may suggest that visual attention training could complement traditional therapies in improving cognitive control efficiency (61, 62). However, substantial further validation is required before any translational application can be considered.

### 4.6 Limitations and future directions

Several limitations should be acknowledged. First, the sample size was modest, which limits statistical power and may reduce the stability and generalizability of both the connectivity and classification findings. Small samples can increase sensitivity to methodological choices and elevate the risk of overfitting, particularly in machine-learning analyses of neurophysiological data (63, 64). Although cross-validation and permutation testing were used to reduce this risk, they cannot eliminate it entirely. Second, the present study employed a cross-sectional design, which prevents determination of whether the observed neurophysiological alterations represent compensatory adaptations, developmental features, or state-dependent changes associated with depressive symptoms. Third, only the Stroop task was used, meaning that the current findings are specific to conflict processing and response inhibition; future studies should incorporate additional paradigms probing working memory, sustained attention, and emotional regulation. Fourth, the exclusion of some clinical complexities, such as comorbid anxiety and longitudinal treatment effects, limits the extent to which the findings can be generalized to broader clinical populations.

In addition, methodological limitations inherent to EEG-based connectivity analysis should be considered. PLV reflects synchrony at the scalp level and may be influenced by volume conduction, while providing limited information about directionality or more complex nonlinear interactions (28, 29). The spatial resolution of the standard 10-20 EEG system also restricts precise localization of neural generators and limits inference regarding deeper brain structures involved in mood regulation and cognitive control (65, 66). Future studies would benefit from larger multi-site cohorts, longitudinal designs, higher-density EEG recordings, source-level connectivity analyses, and multimodal imaging approaches. It may also be valuable to combine resting-state and task-state features in future classification models, as these may capture complementary aspects of neural dysfunction in adolescent MDD (67).

## 5 Conclusion

In conclusion, adolescents with MDD exhibited cognitive control deficits during the Stroop task, characterized by prolonged reaction time, reduced P300 amplitude, altered alpha/beta oscillatory activity, and reorganization of task-state functional connectivity. Together, these findings suggest inefficient attentional resource allocation and altered large-scale neural coordination, with reduced reliance on frontoparietal control systems and greater involvement of occipitotemporal regions during conflict processing. The multimodal classification results further support the potential value of task-related neurophysiological features for distinguishing adolescents with MDD from healthy controls under controlled experimental conditions. Overall, this study provides convergent evidence that cognitive control dysfunction in adolescent MDD can be captured at behavioral, electrophysiological, and network levels, and offers a preliminary basis for future research on objective assessment and treatment monitoring.

## Data Availability

The raw data supporting the conclusions of this article will be made available by the authors, without undue reservation.
